# GLP-1 Receptor Agonists Are Associated With Lower Early Revision Risk Compared to Bariatric Surgery Following Total Hip Arthroplasty: A Retrospective Cohort Study

**DOI:** 10.1177/15563316261492267

**Published:** 2026-10-07

**Authors:** Smit Brahmbhatt, Amal Mathew, Seth Shoap, Arun Kumar

**Affiliations:** 1 Penn State College of Medicine, Hershey, PA, USA; 2 Department of Orthopaedics & Rehabilitation, Penn State Milton S Hershey Medical Center, PA, USA

**Keywords:** total hip arthroplasty, bariatric surgery, GLP-1 receptor agonist, obesity, periprosthetic joint infection

## Abstract

**Background:**

Obesity is a major modifiable risk factor in total hip arthroplasty (THA), associated with increased complications. While bariatric surgery has been the standard for massive weight loss, glucagon-like peptide-1 receptor agonists (GLP-1 RAs) are increasingly used.

**Purposes:**

We sought to answer 2 questions: (1) Are early postoperative complication risks different between THA patients using GLP-1 RAs versus prior bariatric surgery? (2) Do differences persist at mid-term follow-up?

**Methods:**

We conducted a retrospective cohort study using the TriNetX Global Collaborative Network to identify adult patients (≥18 years) who underwent primary THA (current procedural terminology code 27130) between January 2015 and December 2025. Patients were divided into 2 cohorts: those with an active prescription for a GLP-1 RA (semaglutide, dulaglutide, liraglutide, tirzepatide, exenatide, albiglutide, or lixisenatide) within 18 months prior to the index THA procedure (N = 6877) or those with a history of bariatric surgery (sleeve gastrectomy, Roux-en-Y gastric bypass, adjustable gastric banding, and biliopancreatic diversion) within 18 months prior to the index THA procedure (N = 3960). Propensity-score matching (1:1) balanced cohorts on demographics and comorbidities. Outcomes included revision THA, dislocation/instability, PJI, wound complications, emergency department (ED) visits, and readmission at 1 to 90 days and 91 days to 1 year postoperatively.

**Results:**

Within 90 days, the GLP-1 RA cohort had lower risks of revision, PJI, ED visits, readmission, wound dehiscence, and dislocation. At mid-term follow-up, GLP-1 RA patients continued to show lower risks of PJI, readmission, and all-cause revision.

**Conclusion:**

Preoperative GLP-1 RA use was associated with lower early and mid-term complications compared to bariatric surgery, suggesting a potentially safer option for pre-THA weight optimization.

**Level of Evidence:**

Level III: retrospective cohort study.

## Introduction

Obesity represents a significant and modifiable risk factor in total hip arthroplasty (THA), as patients with obesity demonstrate substantially higher risks of complications and revisions compared to non-obese patients.^1^ Meta-analyses have demonstrated that patients with obesity face increased odds of deep infections (odds ratio [OR] 2.71), dislocations (OR 1.72), revisions (OR 1.44), and readmissions (OR 1.37), with risks escalating in morbidly obese patients.^1^ The risk of periprosthetic joint infection (PJI) increases exponentially beyond a body mass index (BMI) threshold of 37.4 kg/m^2^, and patients at BMI 50 kg/m^2^ face a 2.55-fold increased risk compared to those at the threshold.^2^

Traditional weight loss strategies have centered on bariatric surgery, which effectively reduces weight and associated comorbidities. However, bariatric procedures carry inherent surgical risks and frequently result in nutritional deficiencies that may adversely impact bone health and soft tissue healing.^3,4^ Common deficiencies following bariatric surgery include vitamin D, calcium, iron, vitamin B_12_, folate, and protein, which can lead to osteoporosis, impaired wound healing, and anemia.^5-7^ While recent evidence suggests that bariatric surgery prior to THA may not lead to worse surgical outcomes, some studies report increased risks of dislocation, revision, and infection compared to matched controls.^1,8,9^

Glucagon-like peptide-1 receptor agonists (GLP-1 RAs) have been shown to promote weight loss primarily by suppressing appetite through central nervous system pathways, delaying gastric emptying, and reducing food cravings by altering the brain’s reward system.^10,11^ These combined effects decrease caloric intake by up to 39%, resulting in up to a 24% reduction of total body weight.^12,13^ Unlike bariatric surgery, GLP-1 RAs do not impair nutrient absorption, but reduced food intake may lead to insufficient intake of nutrients such as iron, calcium, and vitamins, especially when daily caloric intake is less than 1200 kcal.^13^ There have also been concerns regarding lean body mass, with up to 40% of total weight loss being lean mass. However, overall body composition generally improves.^12,13^ Notably, GLP-1 RAs have not been linked to the severe malnutrition seen after bariatric procedures, suggesting a potentially more favorable nutritional profile in the perioperative setting.

Beyond their weight loss effects, GLP-1 RAs demonstrate multisystemic properties including anti-inflammatory effects, improved endothelial function, and potential benefits for wound healing.^14,15^ Recent surgical literature suggests that perioperative GLP-1 RA use is associated with reduced postoperative complications, including lower rates of readmission, wound dehiscence, and infection across various surgical procedures.^14,16,17^

Therefore, we asked: (1) Are there differences in the risks of early (1-90 days) postoperative complications between THA patients who utilized GLP-1 RAs for weight management versus those with a history of bariatric surgery? (2) Do differences in complication risks persist into the mid-term postoperative period (91 days-1 year)?

*AI Use Disclosure*: We used OpenEvidence to conduct a literature search and Google Gemini Notebook to organize raw data into tabular format. We reviewed and verified all content and take full responsibility for the final article.

## Methods

This retrospective, population-based cohort study was performed using the TriNetX Global Collaborative Network, a global federated electronic health records database containing de-identified patient information from 170 large healthcare organizations (HCOs). The available data included demographics, diagnoses (based on the International Classification of Diseases, 10th Revision, Clinical Modification [ICD-10-CM] codes), procedures (coded in the ICD-10, Procedure Coding System or current procedural terminology [CPT]), medications (RxNorm), laboratory values, and genomics.^18^ The authors had access to the TriNetX database strictly through a federated, web-based query interface. Access was limited to defining cohorts, establishing propensity-score matching, and executing statistical analyses on de-identified, aggregated patient data. Given the use of de-identified data, the requirement for informed consent and IRB approval was deemed exempt. Data cleaning was performed internally by the TriNetX platform’s automated processes. This includes mapping data from participating HCOs, using standard clinical terminologies, and filtering missing data prior to querying and analysis.

We identified adult patients (≥18 years) who underwent primary THA (CPT code 27130) between January 2015 and December 2025. Patients were divided into 2 cohorts: those with an active prescription for a GLP-1 RA (semaglutide, dulaglutide, liraglutide, tirzepatide, exenatide, albiglutide, and lixisenatide) within 18 months prior to the index THA procedure or those with a history of bariatric surgery (sleeve gastrectomy, Roux-en-Y gastric bypass, adjustable gastric banding, and biliopancreatic diversion) within 18 months prior to the index THA procedure. The 18 month preoperative window was selected to capture patients who were actively engaged in weight management strategies while allowing sufficient time for weight stabilization. Patients who had prior bariatric surgery were excluded from the GLP-1 RA cohort, and patients who had prior GLP-1 RA use were excluded from the bariatric surgery cohort. To ensure elective primary THA, patients who had femoral or pelvic fractures within 1 month before THA were also excluded from the study.

To minimize selection bias and confounding, propensity-score matching (1:1) was performed. Cohorts were matched on variables including age, sex, race/ethnicity, BMI, whole blood Hemoglobin A_1c_ (HbA_1c_), serum albumin, diabetes mellitus, primary hypertension, angina pectoris, acute myocardial infarction, acute ischemic heart disease, chronic ischemic heart disease, chronic kidney disease, heart failure, chronic obstructive pulmonary disease, nicotine dependence, age-related osteoporosis, vitamin D deficiency, and anticoagulants. Cohort balance was assessed using standardized mean differences (SMDs), with values <0.1 indicating adequate balance.

The TriNetX Global Collaborative Network contained a total population of 205 403 425 patients at the time of the query. The study period included all eligible index events occurring within the database history up to the analysis date of December 27, 2025. Per standard TriNetX parameters, historical data for index events were limited to a 20 year lookback period. After propensity-score matching, 2640 patients were included in each cohort (Figure 1). Baseline characteristics were well-balanced between groups (Table 1). All comorbidities demonstrated SMD <0.1, confirming adequate matching. Despite matching, overall BMI and HbA_1c_ remained slightly imbalanced, along with serum albumin. However, all categorical bins for these variables were well-balanced. This suggests minor differences within comparable categories rather than clinically meaningful differences between cohorts.

**Figure 1.**
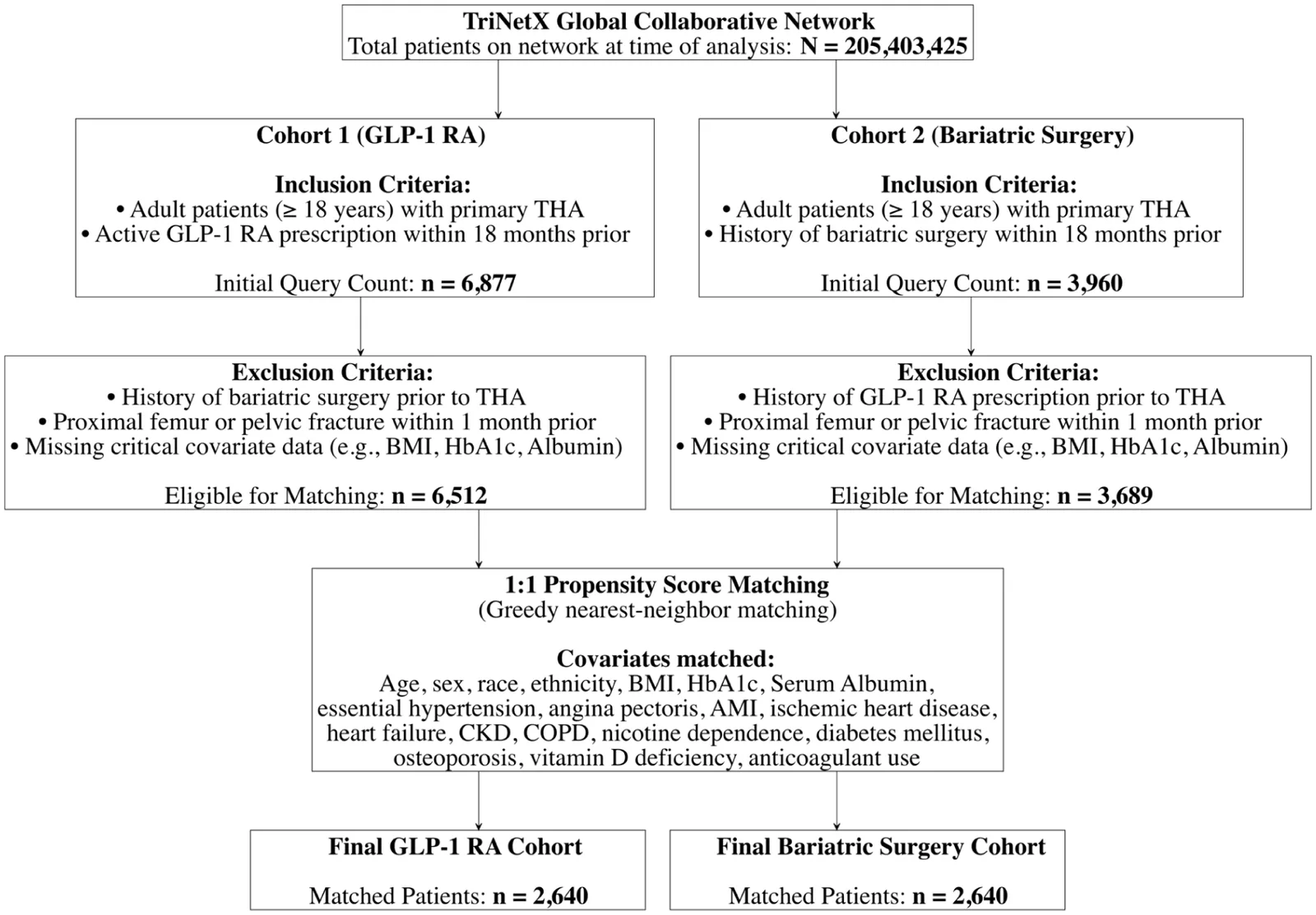
STROBE flowchart of patient selection and propensity-score matching. AMI: acute myocardial infarction; BMI: body mass index; CKD: chronic kidney disease; COPD: chronic obstructive pulmonary disease; GLP-1 RA: glucagon-like peptide-1 receptor agonist; HbA_1c_: hemoglobin A_1c_; THA: total hip arthroplasty.

**Table 1.** Baseline Characteristics of the Propensity-Score-Matched Cohorts.

Characteristic	GLP-1 RA (N = 2640)	Bariatric surgery (N = 2640)	Standardized mean difference	*P* value
Age at index, y (Mean ± SD)	61.4 ± 9.7	61.6 ± 9.5	0.016	.556
Sex, n (%)				
Women	1839 (69.7)	1828 (69.2)	0.009	.742
Men	799 (30.3)	811 (30.7)	0.010	.720
Race, n (%)				
White	2109 (79.9)	2120 (80.3)	0.010	.705
Black or African American	396 (15.0)	380 (14.4)	0.017	.534
Asian	15 (0.6)	15 (0.6)	<0.001	1
Ethnicity, n (%)				
Not Hispanic or Latino	2201 (83.4)	2206 (83.6)	0.005	.853
Hispanic or Latino	86 (3.3)	85 (3.2)	0.002	.938
Body mass index, kg/m^2^ (mean ± SD)	35.6 ± 6.3	34.9 ± 6.4	0.111	<.001
Comorbidities, n (%)				
Essential hypertension	1802 (68.3)	1810 (68.6)	0.007	.813
Diabetes mellitus	930 (35.2)	936 (35.5)	0.005	.863
Vitamin D deficiency	946 (35.8)	962 (36.4)	0.013	.647
Chronic ischemic heart disease	511 (19.4)	519 (19.7)	0.008	.781
Nicotine dependence	372 (14.1)	375 (14.2)	0.003	.906
Chronic kidney disease	291 (11.0)	306 (11.6)	0.018	.514
Age-related osteoporosis	259 (9.8)	273 (10.3)	0.018	.522
Chronic obstructive pulmonary disease	250 (9.5)	256 (9.7)	0.008	.779
Heart failure	240 (9.1)	271 (10.3)	0.040	.149
Medications, n (%)				
Anticoagulants	1310 (49.6)	1301 (49.3)	0.007	.804
Laboratory values (mean ± SD)				
Hemoglobin A_1c_ (%)	5.8 ± 0.9	5.7 ± 0.8	0.139	<.001
Serum albumin (g/dL)	4.2 ± 0.4	4.1 ± 0.4	0.334	<.001

Abbreviations: GLP-1 RA, glucagon-like peptide-1 receptor agonist; SD, standard deviation.

A standardized difference <0.1 indicates a negligible difference between cohorts. Standardized differences for variables without statistically significant *P* values confirm the cohorts were adequately balanced following propensity-score matching.

Outcomes were identified via ICD-10-CM and CPT codes (Supplemental Table 1). Revision THA, periprosthetic joint infection (PJI), dislocation/instability, wound dehiscence, surgical site infection (SSI), surgical debridement, periprosthetic fracture, prosthesis breakage, all-cause readmission, and emergency department (ED) visits were assessed during 1 to 90 days (early postoperative period) and 91 days to 1 year (mid-term period) following the index THA procedure.

### Statistical Analyses

Risk ratios, ORs, and 95% confidence intervals (CIs) were calculated for each outcome. Kaplan-Meier survival analysis was performed with log-rank testing to compare time-to-event probabilities between cohorts. Hazard ratios were calculated using Cox proportional hazards models. Statistical significance was set at *P* *<* .05. All analyses were performed using the TriNetX platform.

## Results

During the early postoperative period, the GLP-1 RA cohort demonstrated significantly lower risks of multiple complications compared to the bariatric surgery cohort (Table 2). Patients who were using GLP-1 RA had lower risks of revision THA (0.7% vs 1.8%; OR 0.40, 95% CI 0.23-0.68, *P* = .001) and PJI (1.3% vs 2.9%; OR 0.43, 95% CI 0.29-0.65, *P* *<* .001). Readmission risk was lower in the GLP-1 RA cohort (0.7% vs 2.1%; OR 0.32, 95% CI 0.19-0.54, *P* *<* .001), as were ED visits (8.5% vs 13.1%; OR 0.62, 95% CI 0.52-0.74, *P* *<* .001). Wound-related complications were also reduced, with wound dehiscence occurring in 1.4% of GLP-1 RA patients versus 2.4% of bariatric patients (OR 0.60, 95% CI 0.40-0.90, *P* = .012). Similarly, surgical debridement was lower in GLP-1 RA users (0.7% vs 1.3%; OR 0.56, 0.32-0.98, *P* = .038). The GLP-1 RA cohort demonstrated a reduction in dislocation/instability (0.6% vs 1.3%; OR 0.50, 95% CI 0.28-0.89, *P* = .017). Risks of periprosthetic fracture and SSI did not differ significantly between groups.

**Table 2.** Comparison of 90 Day Postoperative Outcomes (GLP-1 RA vs Bariatric Surgery).

Outcome	GLP-1 RA (N = 2640), n (%)	Bariatric surgery (N = 2640), n (%)	Risk ratio (95% CI)	Odds ratio (95% CI)	*P* value
Periprosthetic joint infection	34 (1.3)	77 (2.9)	0.44 (0.30-0.66)	0.43 (0.29-0.65)	<.001
Revision THA	19 (0.7)	47 (1.8)	0.40 (0.24-0.69)	0.40 (0.23-0.68)	.001
Readmission	18 (0.7)	56 (2.1)	0.32 (0.19-0.55)	0.32 (0.19-0.54)	<.001
Emergency department visits	224 (8.5)	345 (13.1)	0.65 (0.55-0.76)	0.62 (0.52-0.74)	<.001
Wound dehiscence	38 (1.4)	63 (2.4)	0.60 (0.41-0.90)	0.60 (0.40-0.90)	.012
Dislocation/instability	17 (0.6)	34 (1.3)	0.50 (0.28-0.89)	0.50 (0.28-0.89)	.017
Surgical debridement	19 (0.7)	34 (1.3)	0.56 (0.32-0.98)	0.56 (0.32-0.98)	.038
Incision and drainage/arthrotomy	18 (0.7)	32 (1.2)	0.56 (0.32-1.00)	0.56 (0.31-1.00)	.047
Periprosthetic fracture	16 (0.6)	22 (0.8)	0.73 (0.38-1.38)	0.73 (0.38-1.39)	.329
Surgical site infection	19 (0.7)	24 (0.9)	0.79 (0.44-1.44)	0.79 (0.43-1.45)	.444

Abbreviations: CI, confidence interval; GLP-1 RA, glucagon-like peptide-1 receptor agonist; THA, total hip arthroplasty.

*P* < .05 indicates statistical significance.

During the mid-term interval (91 days-1 year) the GLP-1 RA cohort maintained significantly lower complication risks (Table 3). PJIs remained lower in the GLP-1 RA group (1.2% vs 2.9%; OR 0.41, 95% CI 0.27-0.63, *P* *<* .001), as did readmission risk (0.6% vs 1.3%; OR 0.44, 95% CI 0.24-0.81, *P* = .006). Revision THA was also less frequent in patients who used GLP-1 RA (0.7% vs 1.3%; OR 0.53, 95% CI 0.30-0.93, *P* = .026). No significant differences were observed in dislocation/instability or prosthesis breakage within this period.

**Table 3.** Comparison of Mid-Term (91 Days-1 Year) Postoperative Outcomes (GLP-1 RA vs Bariatric Surgery).

Outcome	GLP-1 RA (N = 2640), n (%)	Bariatric surgery (N = 2640), n (%)	Risk ratio (95% CI)	Odds ratio (95% CI)	*P* value
Periprosthetic joint infection	32 (1.2)	76 (2.9)	0.42 (0.28-0.63)	0.41 (0.27-0.63)	<.001
Readmission	15 (0.6)	34 (1.3)	0.44 (0.24-0.81)	0.44 (0.24-0.81)	.006
Revision THA	18 (0.7)	34 (1.3)	0.53 (0.30-0.94)	0.53 (0.30-0.93)	.026
Dislocation/instability	23 (0.9)	32 (1.2)	0.72 (0.42-1.23)	0.72 (0.42-1.23)	.223

Abbreviations: CI, confidence interval; GLP-1 RA, glucagon-like peptide-1 receptor agonist; THA, total hip arthroplasty.

## Discussion

This retrospective cohort study using a large database found that those taking a GLP-1 RA prior to primary THA surgery had significantly lower risk of both early and mid-term postoperative complications compared to those with a history of bariatric surgery. In response to our first question, we found that GLP-1 RA use prior to primary THA was associated with significantly lower risks of early postoperative complications, including revision, PJI, readmission, wound dehiscence, and dislocation within the first 90 days. In response to our second question, we found that these differences persisted through 1 year, especially with lower risks of PJI, readmission, and revision. These data suggest that GLP-1 RAs may warrant consideration as an option for preoperative weight optimization in THA candidates who are morbidly obese and how they compare with other existing strategies.

Several limitations require consideration. This retrospective database analysis cannot establish causality or account for unmeasured confounders. The TriNetX database, while expansive, lacks granular data regarding GLP-1 RA type, dosing, or duration of therapy, which may influence metabolic outcomes differently. The database also lacks information on patient compliance with nutritional supplementation. Despite propensity-score matching, residual imbalance remained for serum albumin (SMD 0.334), HbA_1c_ (SMD 0.139), and BMI (SMD 0.111). The TriNetX platform does not allow for additional adjustments, but categorical bins for each variable were well-balanced. Additionally, these were the most recent values recorded in patient health records. TriNetX does not allow for extraction of pre-treatment data, so the degree of weight loss could not be determined. A subgroup analysis restricted to BMI ≥40 kg/m^2^ was not performed, as the current cohorts reflect real-world BMI ranges at which both GLP-1 RAs and bariatric surgeries may be utilized. Restricting the population may substantially reduce sample size and statistical power. Future studies are needed to examine whether these findings hold for patients within this BMI range. Selection bias remains a possibility, as patients who choose pharmacological management may differ fundamentally in their health-seeking behaviors or comorbidities compared to those pursuing surgical weight loss. Finally, the 1 year follow-up period, while sufficient for early complications, does not allow for assessment of long-term implant survivorship or late complications.

Beyond infection and wound outcomes, the substantial reductions in readmission (68% at 90 days, 56% at 1 year) and revision risk (60% at 90 days, 47% at 1 year) carry important clinical and economic implications. The leading causes of surgical readmissions (infections, periprosthetic fracture, and dislocations) are precisely the complications that occurred less frequently in our GLP-1 RA cohort, suggesting that the reduced readmissions reflect cumulative protective effects.^19^ Early revision within 90 days also had similar etiologies and was associated with greater healthcare resource utilization.^20^ The sustained reduction in both metrics through 1 year suggests lasting protective effects extending beyond the immediate perioperative period.

The difference in outcomes between these 2 cohorts likely stems from the distinct physiological impacts of GLP-1 RAs versus surgical weight loss. Beyond simple weight reduction and glycemic control, GLP-1 RAs possess pleiotropic anti-inflammatory and immunomodulatory properties.^14,15,21^ This may explain the reduced risks of wound dehiscence and infection observed in our study, supporting recent surgical studies that link active GLP-1 RA prescriptions to improved perioperative recovery.^14,22^ Additionally, meta-analyses specific to arthroplasty have reinforced these effects, showing that GLP-1 RA use was associated with reduced PJI risk at both 90 days (OR 0.73) and 2 years (OR 0.72) postoperatively.^23^

Conversely, the physiological cost of bariatric surgery, particularly malabsorptive procedures, can be notable. The prevalence of secondary hyperparathyroidism and deficiencies in protein, vitamin D, and calcium following Roux-en-Y gastric bypass often leads to accelerated bone loss and compromised soft tissue integrity.^24^ With a documented 21% to 44% increase in fracture risk post-bariatric surgery, the skeletal environment may be less than ideal for arthroplasty fixation.^24^ This metabolic fragility likely contributed to the doubled risk of dislocation seen in our bariatric cohort (1.3% vs 0.6%, *P* = .017), aligning with prior studies highlighting increased instability following bariatric surgery.^3,4^

The timing and nature of weight loss also warrant scrutiny. Recent data suggest that rapid weight loss (3-9 months preoperatively) often increases infection risk, whereas more gradual or stabilized weight loss appears protective.^25^ Bariatric surgery frequently induces profound weight loss in the immediate postoperative period, potentially starving the immune system of the resources needed for surgical recovery. In contrast, GLP-1 RAs facilitate a more gradual weight reduction while simultaneously dampening systemic inflammation.^26^ This suggests that the way a patient loses weight may be just as critical as the amount of weight lost.

Despite the limitations of this retrospective study, our findings have important clinical implications. For patients with obesity considering THA, GLP-1 RAs may represent a safer preoperative strategy compared to bariatric surgery, offering benefits without the nutritional deficiencies, bone loss, and surgical risks associated with bariatric procedures. The expanding indications for GLP-1 RAs, including U.S. Food and Drug Administration approval for chronic weight management in non-diabetic patients, make these agents increasingly accessible for preoperative strategies. However, surgeons should engage in shared decision-making with patients, discussing the risks and benefits of various weight management strategies while recognizing that delaying surgery for weight loss may not improve outcomes, but it could restrict access to beneficial treatment.

In conclusion, this large propensity-score-matched analysis found that preoperative GLP-1 RA use was associated with significantly lower risks of early and mid-term complications following THA compared to recent bariatric surgery. Patients who were in the GLP-1 RA cohort demonstrated lower risks of revision, PJI, wound complications, dislocation, and readmission. These findings suggest that GLP-1 RAs may represent a safer and more effective strategy for preoperative weight optimization in THA candidates compared to bariatric surgery. Future prospective, randomized trials are essential to determine the optimal timing and duration of GLP-1 RA therapy relative to surgery.

## Supplemental Material

sj-docx-1-hss-10.1177_15563316261492267 – Supplemental material for GLP-1 Receptor Agonists Are Associated With Lower Early Revision Risk Compared to Bariatric Surgery Following Total Hip Arthroplasty: A Retrospective Cohort Study Supplemental material, sj-docx-1-hss-10.1177_15563316261492267 for GLP-1 Receptor Agonists Are Associated With Lower Early Revision Risk Compared to Bariatric Surgery Following Total Hip Arthroplasty: A Retrospective Cohort Study by Smit Brahmbhatt, Amal Mathew, Seth Shoap and Arun Kumar in HSS Journal®

sj-docx-2-hss-10.1177_15563316261492267 – Supplemental material for GLP-1 Receptor Agonists Are Associated With Lower Early Revision Risk Compared to Bariatric Surgery Following Total Hip Arthroplasty: A Retrospective Cohort Study Supplemental material, sj-docx-2-hss-10.1177_15563316261492267 for GLP-1 Receptor Agonists Are Associated With Lower Early Revision Risk Compared to Bariatric Surgery Following Total Hip Arthroplasty: A Retrospective Cohort Study by Smit Brahmbhatt, Amal Mathew, Seth Shoap and Arun Kumar in HSS Journal®

sj-docx-3-hss-10.1177_15563316261492267 – Supplemental material for GLP-1 Receptor Agonists Are Associated With Lower Early Revision Risk Compared to Bariatric Surgery Following Total Hip Arthroplasty: A Retrospective Cohort Study Supplemental material, sj-docx-3-hss-10.1177_15563316261492267 for GLP-1 Receptor Agonists Are Associated With Lower Early Revision Risk Compared to Bariatric Surgery Following Total Hip Arthroplasty: A Retrospective Cohort Study by Smit Brahmbhatt, Amal Mathew, Seth Shoap and Arun Kumar in HSS Journal®

sj-docx-4-hss-10.1177_15563316261492267 – Supplemental material for GLP-1 Receptor Agonists Are Associated With Lower Early Revision Risk Compared to Bariatric Surgery Following Total Hip Arthroplasty: A Retrospective Cohort Study Supplemental material, sj-docx-4-hss-10.1177_15563316261492267 for GLP-1 Receptor Agonists Are Associated With Lower Early Revision Risk Compared to Bariatric Surgery Following Total Hip Arthroplasty: A Retrospective Cohort Study by Smit Brahmbhatt, Amal Mathew, Seth Shoap and Arun Kumar in HSS Journal®

sj-docx-5-hss-10.1177_15563316261492267 – Supplemental material for GLP-1 Receptor Agonists Are Associated With Lower Early Revision Risk Compared to Bariatric Surgery Following Total Hip Arthroplasty: A Retrospective Cohort Study Supplemental material, sj-docx-5-hss-10.1177_15563316261492267 for GLP-1 Receptor Agonists Are Associated With Lower Early Revision Risk Compared to Bariatric Surgery Following Total Hip Arthroplasty: A Retrospective Cohort Study by Smit Brahmbhatt, Amal Mathew, Seth Shoap and Arun Kumar in HSS Journal®
