# Supplementary material for: GLP-1 Receptor Agonists Are Associated With Lower Early Revision Risk Compared to Bariatric Surgery Following Total Hip Arthroplasty: A Retrospective Cohort Study
Source: HSS J. 2026 Oct 7:15563316261492267. Online ahead of print. doi: 10.1177/15563316261492267 (PMC13650054; doi:10.1177/15563316261492267)
Supplement: Supplementary file 5 [file sj-docx-5-hss-10.1177_15563316261492267.docx]

**Supplemental Table 1. Outcome Definitions and Corresponding Billing Codes Stratified by Time Period**

| **Time Period Evaluated** | **Outcome** | **Code Type** | **Codes** |
| --- | --- | --- | --- |
| **1–90 Days** | | | |
|  | **Revision THA** | CPT | 27134, 27137, 27138 |
|  | **Dislocation / Instability** | ICD-10-CM | T84.020, T84.021, S73.0, M24.451, M24.452, M24.459, T84.028, T84.029 |
|  | **Emergency Department (ED) Visits** | CPT | 99281, 99282, 99283, 99284, 99285, 99288 |
|  | **Surgical debridement** | CPT | 11042, 11043, 11044, 11045, 11046, 11047 |
|  | **Prosthetic joint infection (PJI)** | ICD-10-CM | T84.51, T84.52, T84.50, T84.59, T84.7 |
|  | **Periprosthetic Fracture** | ICD-10-CM | M97.0 |
|  | **Wound Dehiscence** | ICD-10-CM, CPT | ICD-10-CM: T81.31, T81.32CPT: 13160 |
|  | **Surgical Site Infection (SSI)** | ICD-10-CM | T81.41, T81.42, T81.43 |
|  | **Readmission** | CPT, SNOMED | CPT: 1013699SNOMED: 737481003, 394656005, 32485007, 305349003, 281685003, 304566005 |
|  | **Incision & drainage / arthrotomy** | CPT | 10180, 27030, 26990 |
| **91 Days–1 Year** | | | |
|  | **Revision THA** | CPT | 27134, 27137, 27138 |
|  | **Dislocation / Instability** | ICD-10-CM | T84.020, T84.021, S73.0, M24.451, M24.452, M24.459, T84.028, T84.029 |
|  | **Prosthetic joint infection (PJI)** | ICD-10-CM | T84.51, T84.52, T84.50, T84.59, T84.7 |
|  | **Periprosthetic Fracture** | ICD-10-CM | M97.0 |
|  | **Wound Dehiscence** | ICD-10-CM, CPT | ICD-10-CM: T81.31, T81.32CPT: 13160 |
|  | **Readmission** | CPT, SNOMED | CPT: 1013699SNOMED: 737481003, 394656005, 32485007, 305349003, 281685003, 304566005 |

***Abbreviations****: THA, Total Hip Arthroplasty; CPT, Current Procedural Terminology; ICD-10-CM, International Classification of Diseases, Tenth Revision, Clinical Modification; SNOMED, Systematized Nomenclature of Medicine.*
